# “Midwives are heroes of the country”: qualitative evaluation of a midwifery education program in South Sudan

**DOI:** 10.3389/fgwh.2023.1215405

**Published:** 2023-08-29

**Authors:** Shiromi M. Perera, Guma Patrick Isa, Abdou Sebushishe, Preethika Sundararaj, Megan Piccirillo, Shanell Xia, Amaya Langaigne, Javed Ali, Sara E. Casey

**Affiliations:** ^1^ International Medical Corps, Washington, DC, United States; ^2^ International Medical Corps, Juba, South Sudan; ^3^ RAISE Initiative, Heilbrunn Department of Population and Family Health, Mailman School of Public Health, Columbia University, New York, NY, United States

**Keywords:** midwifery education, South Sudan, sexual and reproductive health, mixed methods, armed conflict

## Abstract

**Background:**

Countries affected by armed conflict have higher maternal mortality than stable settings. South Sudan has one of the highest maternal mortality ratios in the world, with an estimated 789 maternal deaths per 100,000 live births. Long-term socio-political instability has contributed to significant challenges in its health system. To reduce maternal and newborn morbidity and mortality, South Sudan must increase the number of skilled midwives.

**Methods:**

A cross-sectional mixed methods study was conducted in 2022 to assess the midwifery education program at three schools receiving support from International Medical Corps in South Sudan, including in-depth interviews with 15 midwifery school graduates currently working as midwives, their supervisors, 16 school faculty (in dyads), and two Ministry of Health officials; and nine focus group discussions with women clients of graduate midwives.

**Results:**

Participants identified strengths of the schools, including being well equipped with trained and competent teaching staff, competency-based curriculum, including practical training which prepared graduate midwives to apply their skills in practice. Weaknesses of the program included its dependence on donor funding, inadequate mentorship and number of tutors, and insufficient practice for some services due to low client load at clinical sites. Additionally, participants identified challenges affecting midwives' ability to provide good quality care, including lack of equipment and supplies, low client load, low salaries, and insecurity due to conflict. Nevertheless, women in the community appreciated the immense work that midwives do. Midwives were respected by the community at large, and graduates expressed pride and satisfaction in their job, as well as the positive impact they have had in providing critical services to communities.

**Discussion:**

Overall, the quality of the midwifery education program appears to be strong, however gaps in the program and the provision of quality care remain. The findings highlight the need to ensure sustained funding for midwifery education, as well as health system strengthening to ensure midwives can practice their skills. Continued investment in midwifery education and training is critical to reduce high maternal mortality and morbidity in South Sudan.

## Introduction

Investing in midwifery could prevent two-thirds of maternal and newborn deaths globally (1). Midwives are trained to provide a range of sexual and reproductive health services, including management of uncomplicated pregnancies and deliveries, antenatal and postnatal care, and contraceptive services (2). Trained midwives working in collaboration with other medical professionals have been associated with rapid and sustained decrease in maternal and newborn mortality (3).

In 2020, nearly 290,000 women globally lost their lives during and following pregnancy and childbirth, with about 70% of these maternal deaths occurring in Sub-Saharan Africa (4). Countries affected by armed conflict have maternal mortality ratios that are double those found in conflict-free countries (5). Childbirth and pregnancy are the leading causes of death among women and girls in South Sudan (6). The world's newest country, South Sudan has one of the highest maternal mortality ratios in the world, with an estimated 789 maternal deaths per 100,000 live births, a decrease from over 1,300 maternal deaths at independence in 2011 (7). Other health indicators are also alarming: in 2021, the neonatal mortality rate and under-five mortality rate were 40 and 99 deaths per 1,000 live births, respectively (8). A report from the government of South Sudan and UNFPA attributed the country's high maternal mortality ratio to limited availability of quality health care services and the lack of skilled birth attendants (9). An estimated 19% of births in the country are attended by skilled health personnel (10). Shortages of health workers are rampant across Sub-Saharan Africa, with many countries relying on task-shifting in order to improve health care coverage (11). Even so, mid-level health workers such as midwives are severely lacking in South Sudan. At independence in 2011, the country had only 12 fully qualified midwives (12). As of 2019, this had increased to over 600 fully trained midwives, but this is not enough to meet the needs of the South Sudanese population (12). The *State of the World's Midwifery 2021* report highlights the importance of investing in midwives and calls on all governments to increase midwifery education and training (1). Embedding midwives into the health system will advance progress towards Sustainable Development Goal 3—“Good Health and Well-Being” (1).

Since South Sudan gained independence in 2011, long-term socio-political instability has contributed to significant challenges in its health system (13). Economic recession and the government's limited capacity to fund the health system has had a catastrophic impact on the health sector, thus requiring substantial dependence on external sources like international organizations and foreign governments (14). Ongoing conflict that re-started in 2013 caused widespread destruction, a shortage of healthcare workers, and limited health infrastructure, further contributing to these challenges. Additionally, 50% of the South Sudanese population live below the poverty line, and more than 80% of the population live in rural areas with poorly developed infrastructure such as roads and health facilities (15). It is imperative for the country to develop a sustainable health service delivery system and increase adequately trained health personnel, such as midwives, to address the needs of the people.

Since 2008, International Medical Corps (IMC) has co-managed and supported three midwifery schools in South Sudan: Juba College of Nursing and Midwifery (JCONAM), Kajo Keji Health Sciences Institute (KKHSI) and Wau Health Sciences Institute (WHSI). The schools offer a 3-year midwifery diploma program that includes research, management, and leadership training. The diploma curriculum meets the standards on essential competencies established by the International Confederation of Midwives and includes basic emergency obstetric and newborn care (BEmONC). The schools also offer an enrolled midwifery program, which is a 2.5-year certificate program, similar to the diploma program without the research, management, and leadership training. Various pedagogical approaches are utilized to build the skills needed to be successful midwives. Through this education program, IMC has sought to contribute to reductions in maternal, neonatal, and child morbidity and mortality in South Sudan by increasing the number of trained midwives in the country. IMC, with donor funding, provides funding for faculty, scholarship students, resources, and facilities. Since the start of the program, 472 midwives have graduated from the three IMC-supported schools.

An outbreak of violence in 2016 temporarily closed all three schools. KKHSI experienced severe loss, with the destruction of property and equipment. As a result, the entire school, including staff and students, was relocated to Juba for their safety. In Juba, they shared the same premises with JCONAM, leading to considerable issues with space and resources. In August 2022, KKHSI moved back to the original Kajo Keji property.

In 2022, IMC, in collaboration with the Reproductive Health Access, Information and Services in Emergencies (RAISE) Initiative at Columbia University conducted a cross-sectional mixed methods evaluation of the midwifery education program to determine its strengths and weaknesses. The evaluation sought to understand perceptions of midwife graduates and their current supervisors, key stakeholders, and South Sudanese women who received care from the graduates. This manuscript focuses on the qualitative findings; quantitative results will be published elsewhere.

## Materials and methods

The evaluation of the midwifery education program was conducted at the three midwifery schools receiving IMC support: JCONAM, KKHSI, and WHSI. Key informant interviews were conducted individually or in dyads or triads with stakeholders involved in the midwifery education program (Table 1). These included principals, teachers, and clinical preceptors from the three schools and Ministry of Health (MOH) staff in Juba involved in the national midwifery education program.

**Table 1:** Number of key informant interviews.

	JCONAM	KKHSI	WHSI	Total
Principal	–	1	1	2
Teachers (Dyads)	2	1	1	4
Clinical preceptors (Dyads or triads)	2	–	2	4
Ministry of health	–	–	–	2
Total key informant interviews	–	–	–	12

In-depth interviews were conducted with 15 midwife graduates and their supervisors working in Juba, Wau or Malakal at the time of data collection (Table 2). Kajo Keji was still insecure at the time of the study; therefore Malakal was identified as an alternative location since IMC has presence in the area and many midwife graduates were working in the area. Focus group discussions were conducted with female clients of a subset of these midwife graduates. Graduate midwives were purposely selected from a list of graduates provided by the schools whose current job showed they were working in Juba, Wau or Malakal. Respondents were selected to ensure a mix of schools, graduation dates, and sex. Interviewers contacted selected graduates to explain the study and ask if they were willing to participate, and if the interviewer could also speak to their current supervisor about their work. Finally, the graduates were asked to purposely identify a group of women from the local community who had used their midwifery services for a focus group with a goal of completing nine focus groups.

**Table 2:** Number of in-depth interviews and focus group discussions.

	Juba	Wau	Malakal	Total
In-depth interviews	10	10	10	30
Supervisors	5	5	5	15
Midwife graduates	5	5	5	15
Focus group discussions	3	3	3	9

### Data collection

Four interviewers who were themselves midwives (three females and one male) participated in a five-day training covering qualitative methods, research ethics and the study instruments. The teams completed data collection in Juba first, and then the team split in half with two interviewers traveling to Wau and two to Malakal for further data collection. Interviews with graduates, supervisors and key informants were mostly conducted in English. Focus group discussions were conducted by female interviewers primarily in Juba Arabic. Interviewers did not interview faculty or preceptors from the school they attended. Interviews took 30–45 min and focus group discussions lasted 50–80 min. Data collection took place in March and April 2022.

### Analysis

All interviews and focus groups were transcribed, and if needed, translated to English. The English transcripts were clarified with members of the research team in South Sudan. Using an inductive approach, researchers created codebooks for each type of respondent through an iterative process. After the codebooks were finalized, transcripts and codebooks were uploaded into Dedoose (16). All focus group discussions were double coded while interview transcripts were coded by one researcher, with a second researcher coding 20% of the transcripts. When discrepancies arose, they were discussed and resolved until inter-rater reliability was achieved. When coding was completed, researchers conducted thematic content analysis to identify the main themes.

### Ethical considerations

Before the interviews and focus group discussions, verbal informed consent was obtained from all participants. Interviews and discussions were audio recorded; no participant names were included in transcripts. Only members of the research team had access to the recordings and transcripts. The Institutional Review Board of Columbia University and the Ministry of Health for the Republic of South Sudan determined the study to be exempt.

## Results

Results were categorized into six major themes: strengths of the midwifery education program, areas identified for strengthening, effects of the midwifery education program, service delivery challenges, community women's views, and perceptions of male midwives.

### Strengths of the midwifery education program

Participants described the schools as well-equipped to train competent and skilled midwives given the high quality and standards of the faculty. Teachers explained that IMC-supported schools benefited from well trained and competent staff, such as teachers and clinical preceptors. Teachers were described as skilled and confident in their abilities and were known to not only teach, but also shape student's attitudes through mentorship. Several teachers found it rewarding to share their knowledge and mentor students. They described seeing their graduates applying the knowledge and providing good quality services when they visited health facilities as a source of pride. In addition, a few teachers explained how their teaching expands beyond the classroom and their own students when they found themselves teaching students and staff from other schools while at clinical sites.

*“Having been in the private sector, and also having seen what takes place in government- owned and government- run institutes which provide midwifery trainings, you can realize that this midwifery training by IMC, at least it is better facilitated. This is one of the strengths, [it is] better facilitated, you have good well-trained staff, well- trained clinical instructors, competent tutors! I could say in summary that competent staff is one of the strengths, they are well remunerated, compared to others and the graduates that are produced are of good quality because of these factors.” (Teachers, Dyad 3).*

Many respondents referred to the competency-based approach to teaching and the practical component of the curriculum as a strength of the midwifery education program. Practice at clinical sites allowed midwifery students to refine their knowledge and attitudes, while practicing their skills.

*“We look at the curriculum and then we look at how best we allocate [students] according to what they have covered in theory. …and then before they're taken to the clinical [sites], once they”ve finished their first theory, we evaluate them … we evaluate them to look at how best they understood the class teaching and when they are evaluated then we can see the level of performance that they have before we take them to the clinical area. … So we give them that time to learn a lot. You demonstrate and then after that you allow them to do a return demonstration so that you can know the level of competencies.” (Teachers, Dyad 1).*

Clinical preceptors explained the process of successful practical training, which involved close monitoring and providing step-by-step guidance.

*They [students] will be given time to correct their mistakes, but you are still in the learning stage, and this will be between [the student] and the monitor. But these students are good, if they make a mistake and you tell them, they correct the mistake and are happy because you told them the mistake and you help in correcting it.” (Clinical preceptors, Dyad 4).*

Graduates of the midwifery schools explained that the practical component was a vital part of their education, providing skills they were prepared to apply in practice after graduation.

*“What made me a successful midwife was the practical clinical attachment that I had. They gave me exposure and much experience, even when I was still a student, plus the tutorship that I had. So, the combination of the two made me the midwife I am today.” (Male midwife 14).*

Graduates reported feeling prepared to provide a range of midwifery services upon graduation, with family planning, antenatal, and labor/delivery care the most frequently mentioned. The majority of supervisors agreed, indicating that their midwife was well-prepared for the work following their formal training. Most supervisors noted that midwives provided a range of care, including family planning and safe delivery. Midwives also participated in community education to raise awareness about the importance of antenatal care, fulfill administrative tasks, and facilitate referrals to and from the facility.

For a select number of students who could not afford schooling, IMC provided financial support (with donor funds) for tuition and accommodation. Several faculty mentioned this support as a strength.

*“To the students actually they benefited because some students they can't pay school fees going to the university, so their parents are not able to do that. So at least they got free education, they have graduated and they are working. And by working you are able to support your mothers, your parents, in case they are not married so this one is already the benefit for the student.” (Principal 2).*

### Areas identified for strengthening

Graduates described the services that they felt least prepared to provide, with complicated deliveries most frequently mentioned, along with newborn care, care for survivors of gender-based violence, and post-abortion care. Supervisors concurred, mentioning weaknesses in manual vacuum aspiration (MVA) and subcutaneous depot medroxyprogesterone acetate (DMPA-SC) administration as well as documentation and clinical register maintenance. While graduates generally reported being well-prepared to provide family planning, they mentioned needing additional practice and training on long-acting methods, such as intrauterine device (IUD) placement and removal, due to low client load during their training.

*“In family planning, like this IUD, unless supported by another, how to insert, I cannot manage alone.” (Female midwife 15).*

While the practical component of their training was consistently identified as a critical strength of the training, graduates reported some gaps, including insufficient time, mentorship, and clients to practice on. Supervisors also mentioned that the practical components of the midwifery program were insufficient at times as the midwives did not always have the opportunity to put theory into practice during their training.

*“We came and realized that sometimes the number of students was more than the patients. So, any client that comes to the facility, if someone grabs them already, then other students will not get…there is lack of patients” (Female midwife 4).*

Although overall satisfaction with the faculty was high, some participants identified the need for additional and better trained clinical preceptors and tutors. Several graduates discussed weaknesses with some tutors and preceptors that supported their studies, primarily when they were too few, had high turnover, or limited skills. Teachers also suggested that there were too few preceptors at clinical sites to guide the many students for practical training.

*“The only weakness I have seen…is the inconsistency of the tutors. We can have a tutor today, tomorrow, [but] maybe after some months that tutor will be out…although the tutor was not competent enough and the replacement was competent enough, still we felt that was not good and that was a weakness of the institution.” (Male midwife 14).*

Many teachers also worried about the insufficient number of tutors to properly teach large class sizes. Over the previous six years, the number of tutors was reduced due to budget cuts and nationalizing of staff positions. Teachers expressed concern over the reduced quality of teaching and being unable to provide sufficient attention to all students. A few teachers felt that they were doing “double work” to help with the shortage. The MOH respondents suggested that the shortage of qualified teachers and preceptors resulted in some students being supervised and taught by untrained personnel at the clinical sites.

Key informants described the midwifery education program's dependence on donor support and funding issues over the past few years as a substantial weakness. Budget cuts impacted all three schools, resulting in decreased financial support and scholarships, reduced number of staff, and fewer students recruited into the program. The staff pointed out that the government and other stakeholders had no clear plans for sustainability, nor sufficient political will to sustain the school once funds depleted. A few teachers also mentioned low salaries, especially for those employed by the MOH, as particularly demotivating when they were asked to increase their workload following staffing cuts.

*“If you look at these three schools you are doing this survey on, these schools were fully supported by the NGO. So, currently, funding has ended and ending the funding means, there is no more hope again because the Ministry [of Health] has not yet stepped in. So, that one I see it as one of the weaknesses, meaning our government will never look at what will happen after the NGOs leave.” (Principal 1).*

MOH staff explained that the MOH was unable to provide much support to the program given its low budget.

*“The program is ongoing but it's stagnating. It's stagnating. When we had the training, it was donor supported. When there is no donor support, that is where the problem is. The other parties, the government has not put in funds to take care of these midwifery trainings…Because the budget of the Ministry of Health was very low.” (Ministry of Health 1).*

Participants also described the impact of conflict on the education program, with each school closing for periods of time when students and staff could not safely come to school. Since these closures, MOH staff explained the importance of working with principals to develop contingency plans to ensure the schools' continued operation.

*“When there's insecurity that comes out, like in 2016, when the outbreak of civil war in South Sudan broke out…there's no teaching. The activities were locked down. For around 3 months, there were no other activities that were running.” (Teacher, Dyad 4).*

### Effects of the midwifery education program

Teachers described that South Sudan had few midwives prior to independence in 2011, a grave problem until IMC and other partners began supporting midwifery education in the country. Teachers perceived a considerable decrease in maternal and neonatal mortality in the last few decades in South Sudan, partly attributing this reduction to the education program and the increase in the number of midwives in the country.

*“You know, when the census was done, the maternal mortality [ratio] was 2054 at that time in 2008. But now when you compare the maternal mortality at that time and this time, now we have [a maternal mortality ratio of] 789. So, there's a great improvement that has occurred. And why did it come like that? Because there are midwives outside, they’re already serving. At least, they’ve done their best. And not only midwives alone, also nurses, to reduce maternal mortality. … That's why the maternal mortality ratio has gone down. And we hope that it will continue to go down until zero if possible.” (Teacher, Dyad 1).*

Teachers and clinical preceptors explained that midwife graduates worked all over South Sudan, even reaching remote villages to provide much needed services to marginalized communities. Graduates described how their training was relevant to the communities they served as it filled a gap in the existing health services. They reported increases in health facility deliveries and family planning use—a change they credited to their midwifery education and training. Additionally, new midwives were seen to bring “updated” knowledge from their training that they could teach to current staff at facilities.

*“The most important thing about this program is the fact that these mothers are being attended to by skilled, trained midwives. Skilled ones. And, when these mothers are being attended to, it means a lot. You find that she has less chances of getting in this kind of risk during pregnancy and so forth. So, you find that this program it has been positive in the community, especially for mothers.” (Teacher, Dyad 1).*

*“Yes, [the services I was trained in are] relevant because it's helping people… For family planning, it's helping mothers now to delay instead of just rapidly deliver [again]. Also, for mothers who are pregnant they go for antenatal care, for focused antenatal care. Instead of staying at home and delivering from home… [they know] the importance of delivering in the hospital. It's very relevant.” (Female midwife 13).*

Similarly, supervisors also noticed the impact of the work done by midwives. Due to an increase in both quality of care and community education, supervisors indicated that more women were now delivering in facilities, as well as coming for antenatal care (ANC) and family planning. A few supervisors also stated that they made fewer referrals to the hospital now that midwives are present.

*“You know at the beginning, people here have a lot of referrals to [Hospital] but since he came here, we have no more referrals now unless when he cannot manage [them]. Like before, this incomplete abortion we did refer them and when he was not there those people used to go to [Hospital], but after we recruited him and brought him here, now it has reduced the level of referral. Most of the mothers are not reaching [Hospital] because he is now here and can provide all these services.” (Supervisor 7, male midwife).*

Almost all supervisors spoke about how the midwives were respected by community members who understood the importance of the work that midwives do. Several participants also noted reductions in women coming in with infections.

*“The community knows the impact of our midwife in this hospital. Let me give you one example. One time due to some political issues, the OPD [outpatient department] and other services were closed. But the community said you can close all other services except the maternity. … We can never accept to close the maternity. So, I mean that's when the community understood that this service is very important.” (Supervisor 2, male midwife)*

*“Actually, there is great improvement compared to the time before those midwives were working. After the graduation, these new students start coming in to practice; there is a lot of changes in every area. Sepsis has gone down, antenatal has increased, deliveries have increased, family planning has increased.” (Supervisor 5, female midwife)*

Midwives largely reported pride and satisfaction in their work, providing services to women and the positive impact these services have on communities.

*“You know working as a midwife, actually in my experience is a great joy, because you are saving lives and at the end of any successful work, you find that you are putting a smile onto the faces of these women who have delivered and to the family. So that joy gives me happiness and it actually motivates me in the work I am doing as a midwife.” (Male midwife 9).*

*“The life challenges that they go through are not comparable to our neighboring countries because we live in a resource constrained country, resource constrained health facilities. … When they go out there, to me, they beat all the odds. They beat the odds. For them to live year in year out and providing services out there, for me they are heroes. They are heroes of the country.” (Teacher, Dyad 3).*

Teachers and clinical preceptors also discussed their pride in being part of the midwifery profession through their roles in this program. They also expressed pride in the midwives they have trained over the years.

*“So when I think of the fact that these people I trained tomorrow would deliver a mother and the mother and the baby would be safe, I feel so proud because I feel that I'm one of those people who are involved in reducing the maternal mortality ratio… I feel proud about that and that is the reason why I keep moving forward.” (Teacher, Dyad 3).*

*“My two hands as a midwife would not have reached the whole country of South Sudan, but through the training of the students my two hands are almost reaching all states of South Sudan. Meaning it is helping women in almost the whole country, so that makes me very proud. I feel so good because if the mother who is deep in the village there can be attended to by my own students, that is my pride.” (Principal 1).*

### Service delivery challenges

Both supervisors and graduate midwives described barriers to providing good quality care. Some of the barriers included commodity stock outs, lack of equipment, insufficient staff, and no dedicated rooms for services such as family planning and post-abortion care.

*“Some of the challenges or barriers could be sometimes we do not have the [resources] that we needed to deliver [services]. Like for example, I'm supposed to be doing BEmONC [basic emergency obstetric and newborn care], but when I came in we did not have the MVA [manual vacuum aspiration] machine. Sometimes we have post-abortion cases to manage so I feel like this is a gap, this is a barrier. I could not do my best. I have the skills and knowledge but the device to perform is not there. That is one. Another is off and on supply of the commodities.” (Male midwife 14).*

Supervisors noted that midwives were not always able to put theory into practice due to low client load and suggested that midwife graduates may lose competency in some skills due to lack of practice. Several supervisors had provided on the job training, and most said that the midwives had received at least one refresher training, often on BEmONC, post-abortion care and family planning. However, nearly all said the midwives would benefit from additional refresher training.

*“For example, if you have trained how to give [family] planning services, at the end of the day you don't have [supplies], so end up doing nothing. So, you may have that skill until it is wasted, and it has no use.… services have to be available to make sure midwives practice what they have learned in school, and they should continue practicing it in their area of work. And that should help to make midwives succeed, because you cannot have the theoretical part and you don't have the practical part.” (Supervisor 9, male midwife).*

Among the midwives, low salaries and insufficient compensation were frequently mentioned as a challenge. While some accepted the low salary because of the importance and impact of the work, others expressed greater concern, including that low compensation may force them to move into other career paths. Several midwives mentioned working as volunteers in a health facility first before being hired. Key informants voiced that there were not always enough jobs, and those that were available, especially with the government, paid little to no salary.

*“The salary I'm getting, the incentive is very little. But the service that I deliver to the community is more than what I am receiving so I am very proud.” (Female midwife 1).*

Insecurity due to conflict was a common challenge mentioned by respondents in all groups. Key informants described insecurity as a challenge. In times of conflict, midwives and other health workers did not work in insecure areas of the country out of fear, leaving these areas without vital services. Insecurity also posed a challenge for both midwives and women to safely reach health facilities, especially at night.

*“I remember when I was working outside of the town, I could not travel due to insecurity issues. So, during such days I would always feel like now I can’t make it to the field but my clients and patients need me more than I could stay here. That created instability within myself whenever I’m blocked from access to my facility where I could be able to interact with my clients and patients. It always created a sense of insecurity and instability within me.” (Male midwife 14).*

### Community women's views

Women in all focus groups described positive experiences with their midwives. Participants expressed their appreciation for the work that midwives do, especially their handling of pregnancy-related care. Women recognized that they and their babies are safer when receiving care from the midwife.

*Participant 1: “[The midwives] helped me with their words or their medicines or with their things, I am happy with them up to when I gave birth successfully.”*

*Participant 2: “Because she gives you good advice; if you go home, you will remember the things that she told you and you will work with it.” (Community women, FGD 1).*

Others described how the contraceptive services provided by the midwives helped them to better provide for their families, educate their children, and space their births. Mothers who had positive experiences with midwives encouraged other women in the community to seek out midwifery care.

*“Or when you are sick also, they must treat you before you are discharged. You cannot be discharged into the community while the baby is sick, and you are also sick. The midwife does all these before they discharge you, so that there is no complication in your body and no complication in the baby's when you go to the community. The midwife does them all, there is nothing she does not do.” (Community women, FGD 5).*

*“If it is in the place of family planning, they give you this medicine, it will be able to protect you. You will be able to work for yourself. If it is business, you will be able to concentrate for yourself. Even with your children, you will be able to work.” (Community women, FGD 3).*

While participants were mostly satisfied with the care received from the midwives, a few participants across groups described negative experiences with them such as being harassed, yelled at, or judged by midwives, or that the midwives had bad tempers. Other negative experiences that women had were related to health system issues, such as lack of materials like maternity kits, stockouts of medication at the health facility, or the lack of capacity for nighttime deliveries. Long distances to the health facility, and cost of or limited availability of transport created barriers to accessing care.

*“All the things are available, but there is no electricity and there is no torch. The midwife is using her phone light.” (Community women, FGD 4).*

*“There is no midwife who delivers people here at night in [the health facility]. … they carry pregnant women to her home. Everyone gives birth there. I do not know why this hospital does not work at night.” (Community women, FGD 6).*

Overall, women recognized that both they and their babies are safer with the midwife's presence. They described how the good work of the midwives and women's positive experiences encouraged other women in the community to visit the midwife. Several noted that more women in their community deliver in the health facility than previously.

*“Before the midwives were brought, we are suffering, children and women are dying, but since the midwives were brought, there is no death.” (Community women, FGD 2).*

*“A person will hear, like this pregnant mother, if she is given drugs and returns to the community with proper understanding of the nature of the work here, she may mobilize people. … So, now all mothers are here, there is no one in the community or nobody who does not want to come here.” (Community women, FGD 5).*

### Perceptions of male midwives

Nine of the 15 midwife graduates interviewed were male. A few described the need to convince their families and friends that midwifery was a good profession for them. One mentioned that his wife appreciated that he now understood what she goes through during pregnancy and delivery.

*“So, the culture in the community I came from, being a male midwife is a shameful profession. Being in my culture, there is no male midwife…you cannot be a midwife when you are a man.” (Male midwife 10).*

The male midwives said that while they faced some pushback or incredulity when they first arrived in a community, community members eventually came to accept them in their role. In the beginning, when some women would refuse (or their husbands prohibited them) to come see them, they described talking to community members and performing their jobs well which ultimately resulted in their acceptance.

*“Yeah, there is that little bit level of resistance but not from many people… Some of them can refuse to be seen by a male midwife but at the end they will always accept.” (Male midwife 14).*

*“You know at first when you come … and the community see the man is the one conducting deliveries. So in the beginning it was not good, we were facing a lot of challenges but when we get people on more awareness that men also can also conduct delivery [as well as] female then we have no problem. We are receiving our clients and they are very comfortable with us.” (Male midwife 2).*

Focus group participants concurred. Some expressed surprise that male midwives existed, but generally, once they overcame their surprise, they appreciated them. Others described feeling uncomfortable in the presence and care of male midwives.

*“Her surprise is that she has never seen men before conducting delivery and caring for women very well like this. But when she stayed here for two days, she sees positive things. A long time ago women are the ones who can conduct delivery, but now it happens, men also they are conductors. So, she was very surprised when she sees a man now conducting the delivery of her daughter.” (Community women, FGD 5).*

*“When a male midwife is the one conducting deliveries in the hospital and examining the mothers, the mother will refuse and say, “I don’t want this man to examine me.” All these things are there, they discourage pregnant mothers. You may not wish to return, coming back to the hospital, because you are confused.” (Community women, FGD 5).*

## Discussion

Our findings suggest that the overall quality of the midwifery education program is strong. The program was described as high quality and well facilitated. Teachers at the schools were committed to their students. Midwife graduates felt prepared to provide most midwifery services, with their supervisors in agreement about their capabilities. The competency-based approach and practical learning component of the midwifery education program were seen as vital. When midwives are educated to international standards, they can provide a full scope of comprehensive interventions and 80% of maternal deaths could be prevented (17). Participants from our study also reported increased use of midwifery services, including safe delivery, ANC, and contraception. They noted that more women were now delivering in facilities and coming in for ANC and family planning because of the midwives' work. Evidence shows that increased healthcare worker density is associated with increased use of ANC and facility birth (18). Delivery in a health facility with a skilled health worker and good quality ANC contributes to reducing maternal and neonatal mortality. Midwife graduates and teachers expressed great pride in their work, similar to findings from midwives in South Africa who reported that their work boosted their self-esteem (19), and in Mozambique who felt empowered by “saving lives, giving hope, helping people, and having the sense that their work was meaningful” (20).

Despite the overall positive perceptions of the education program and the work done by midwives, participants identified a few areas for improvement. Midwife graduates mentioned feeling less prepared to deliver services such as complicated deliveries, newborn care, care for survivors of gender-based violence, and post-abortion care. Graduates mentioned needing additional practice and training on procedures such as IUD placement and removal due to low client load. This finding is comparable to that of Afghanistan, where a dearth of caseloads in health facilities made it difficult for midwives to maintain their skills (21). It is imperative to ensure that midwives receive enough practical training on a range of services and retain competency after graduation. Providing midwives with refresher training and opportunities for clinical simulation of emergencies and rare cases can be an effective technique to enhance skill performance (22). Moreover, supportive supervision of midwives also plays a pivotal role in improving the quality of care (23). One study with village midwives in Sudan found that consistent and sufficient supervision in conjunction with follow-up improved their skills and knowledge (24).

Another area for improvement identified by the participants was the shortage of qualified preceptors which resulted in some midwife students being taught by untrained personnel at clinical sites. Experienced preceptors play a crucial role in clinical education by providing students with the opportunity to become immersed in their future roles as midwives (25). Challenges to preceptorship in countries across Africa include preceptors who are young and inexperienced, high turnover contributing to inadequate numbers, demanding workload and limited tutoring competence (26). Clinical sites must ensure an adequate number of qualified preceptors are employed so that students can learn and effectively put theoretical knowledge into practice. The program should provide additional training to clinical preceptors to ensure they have the skills to effectively supervise students.

In addition to weaknesses in the education program, midwife graduates also described challenges they faced to practice what they have learned in their postings after graduation. For example, midwife graduates and supervisors mentioned commodity stock outs and lack of equipment as a barrier to the delivery of good quality services. Insufficient medical supplies and lack of equipment have also been observed elsewhere in South Sudan (27, 28). According to Jones et al., procurement and supply chain management of essential medicines was a highly difficult task in South Sudan where the MOH is responsible for supplying essential medicines to healthcare facilities and operates on a “push system based on forecasting” rather than one based on demand, which proved to be unresponsive to the actual needs of the facilities (27). Further, the health sector in South Sudan is critically underfunded, with only 2.9% of the national budget allocated to the sector in 2012–2013 (15) and dropping down to 1.08% in 2019–2020 (29). Key informants described the dependence on donor support and funding as a weakness of the education program. With the decreased allocation of funds, the country is straying further from achieving the Abuja Declaration spending target of 15% of the national budget for health (29). Key informants described the dependence on donor support and funding as a weakness of the education program. This study was conducted during a time when IMC was experiencing challenges with funding for the midwifery education program; however renewed donor funding has since been obtained, addressing at least some of the issues highlighted by teachers. South Sudan's health system continues to rely heavily on donor aid, with non-governmental organizations providing an estimated 70% of health services (15). Decision-making and power over the use of funds often lie with the donor, whose interests may not always align with the government (30). Innovative approaches to health system strengthening and MOH capacity building are needed alongside improved governance and accountability mechanisms and increased national health funding (14). Advocacy to the MOH and other relevant Ministries is needed to obtain a stable stream of funding to sustain the education program.

Midwives frequently mentioned low salaries and insufficient compensation, and several had to work as volunteers. Despite the high need for midwives in South Sudan, public funding is inadequate to pay them. Similar findings of low salaries were reported in a study among maternal and child health providers, including midwives, who reported a monthly salary of as little as 300–500 South Sudanese pounds (US$2.30–3.80) when working at state level or 500–700 South Sudanese pounds (US$3.80–5.40) at tertiary level (28). Inadequate compensation was associated with decreased motivation, poor performance and loss of staff (28). Midwives across Africa and other low income countries report wages that barely meet basic needs and standard of living (31). Additionally, some midwives from our study have expressed concern about low salaries, leading them to consider changing careers. Low salaries were the most common factor contributing to high rates of nurses and midwives in Ghana expressing intention to quit their jobs (32). Not only should the budget allocation for the health sector be increased, but it is also crucial to ensure that the funds are utilized to provide paid positions for trained midwives and that they receive appropriate compensation.

Insecurity due to conflict was mentioned as a challenge by participants in all groups interviewed. Classes were suspended because the three schools had to close for periods of time when it was unsafe for students and staff. Contingency planning is important to reducing disruptions to education and training, which was also mentioned by the MOH staff. Insecurity also affects both providers and women seeking healthcare, especially at nighttime. The most rural areas are likely deprived of skilled providers due to fear and lack of security. Safety is often a major concern, particularly among providers who are young unmarried women, preventing them from being able to provide 24 hour quality care due to the risk of violence and sexual harassment (23, 31). For example, midwives in South Africa and Uganda have reported physical attacks when attending home births or leaving work late at night (31). As seen in other studies, insecurity was also a commonly reported barrier to women accessing maternal healthcare services (28, 33–36). Violence during times of conflict often targets civilians and health workers, causing great harm and even death (28, 37). To increase accessibility and safety, health facilities should consider adding security measures for both providers and clients.

Notably, our findings demonstrated that midwives were more than just providers of care, they were respected leaders and important members of the community. This is contrary to the poor status and image of nurses and midwives found in an earlier study in South Sudan when traditional birth attendants worked as “midwives” in health facilities and were viewed as unskilled and uneducated (15). Cultural influences may also shape the view that assisting childbirth is considered unskilled labor and inherently “women's work” (31). Additionally, many ethnic groups in South Sudan are patriarchal where men hold authority on all aspects of the family and society, while women are viewed as inferior (38). Due to this, early marriage and negative attitudes toward female educational attainment are common which contribute to low school enrollment of girls (39). Only 10.9% of women in South Sudan have completed upper secondary school (40) which poses a challenge to identifying sufficient female students for midwifery schools since eligibility criteria include the completion of secondary school (15). Although midwifery remains a profession that is almost entirely represented by women globally (31), the midwifery program in South Sudan has enrolled a sizeable number of male students. Some community members who have received care from graduate midwives were not aware that male midwives existed. However, many of those who did see a male midwife came to appreciate the work that they do while others described feeling uncomfortable having a male midwife provide care. Findings from a study in South Sudan investigating the community's preference for the gender of midwives revealed similarly mixed results with some participants preferring male midwives due to their considerate and prompt work, while others expressed shame at undressing and discussing sex with male midwives (41). Increasing the number of male midwives can help address the shortage of midwives in the country, while also challenging gender norms in a society where midwives may be viewed as inferior.

### Study limitations

Our study was subject to several limitations. We interviewed midwife graduates and supervisors who were accessible in terms of location and security. We selected three locations for data collection where we knew many graduates would be found: Juba, Wau, and Malakal. Kajo Keji was not safe for our research team to visit at the time this study was conducted. The midwife graduates and supervisors we interviewed were limited to those working in health facilities that were accessible given security and distance from one of the three towns visited, excluding very rural locations. In addition, when interviewing women from the community who used services at the health facilities, it is possible that they spoke about experiences with midwives other than the one who was interviewed as they may have engaged with multiple midwives during their care. Although interviews were not conducted by IMC staff, the interviewers were hired by IMC which may have resulted in courtesy bias wherein respondents may have provided favorable responses to the researchers.

## Conclusion

Overall, graduates of the midwifery education program are working around South Sudan, delivering positive impacts in their communities. Most expressed pride in their work, and their contribution to their communities—despite the many challenges they face. The country's health system requires strengthening, increased budget allocations to the health sector, and security and protections put in place for providers and clients alike. As one teacher mentioned, midwives are heroes that the country needs. Continued investment in midwifery education and training is needed as midwives are critical to reduce high maternal mortality in South Sudan, and other countries similarly affected by conflict.

## Data Availability

The raw data supporting the conclusions of this article will be made available by the authors, without undue reservation.
